# Propagation of antibacterial cold atmospheric pressure plasma through small-bore tubing

**DOI:** 10.1371/journal.pone.0328375

**Published:** 2025-08-21

**Authors:** Elanie F. Briggs, Bhagirath Ghimire, John A. Mayo, Maryellen S. Kelly, Kunning G. Xu, Tatyana A. Sysoeva

**Affiliations:** 1 Department of Biological Sciences, The University of Alabama in Huntsville, Huntsville, Alabama,; 2 Department of Mechanical and Aerospace Engineering, The University of Alabama in Huntsville, Huntsville, Alabama; 3 Healthcare of Women and Children Division, School of Nursing, Duke University, Durham, North Carolina; 4 Department of Urology, Duke University, Durham, North Carolina; Universidad Autonoma de Chihuahua, MEXICO

## Abstract

Cold atmospheric pressure plasma (CAPP) is well documented to have microbicidal properties including against non-pathogenic *Escherichia coli*. In this feasibility study, a semi-quantitative method was developed to measure bactericidal activity of argon-CAPP (Ar-CAPP). Ar-CAPP susceptibility then was tested for a variety of clinically relevant *E. coli* strains, both uropathogenic *E. coli* (UPEC) and strains resistant to multiple antibiotics. All strains tested were found similarly susceptible to the antibacterial effects of Ar-CAPP. Next, methods were developed to propagate Ar-CAPP through defined lengths of plastic tubing with internal diameters ranging from 0.8 to 3.5 mm. Ar-CAPP propagated throughout all sizes of tubing tested, with visible plasma plumes exiting from the distal ends of the tubing. Bactericidal effects of the plasma efflux were tested against *E. coli*. Only partial loss of bactericidal activity was seen in tubing efflux plasma compared to control plasma jet plume unobstructed by tubing. Future work will develop bacterial biofilms on the internal surfaces of tubing to determine whether Ar-CAPP will ablate biofilms or kill biofilm bacteria. If successful, Ar-CAPP could further be tested for feasibility of disinfection of urinary catheters and other re-useable insertable medical devices in at-home and hospital settings.

## Introduction

Plasma, as a state of matter, is defined as an ionized gas containing significant amounts of electrons, ions, and reactive species. Cold plasmas are produced in the laboratory by applying high voltage to a metal electrode in the presence of noble gases such as argon or helium. In ambient environment, a CAPP will produce ultraviolet (UV) radiation and reactive oxygen (ozone, hydrogen peroxide) and nitrogen (nitrate, nitrite, etc.) radical species as well as electrons and ions [1–4]. Target microbial cells, stressed by overloading with charged particles and reactive oxygen and nitrogen species, will suffer lipid peroxidation, disruption of cell walls and membranes, and degradation of proteins and nucleic acids, leading to cell death [5,6]. CAPP is relatively easy to produce using commercial components, thus may be a suitable low-cost disinfection method for at-home use as well as in manufacturing or hospitals. CAPP is useful in dermatology [7], and portable, hand-held devices for cosmetic treatment are commercially available (e.g., LUXE Plasma Pen, https://luxeplasma.com/products/luxe-plasma-pen).

Previous studies have noted that CAPP does not cause erosion of the surface of soft materials similar to urinary catheters [2,8–10]. CAPPs are microbicidal against viruses, fungi, and numerous bacterial genera [4,11]. Susceptible bacterial genera include those containing pathogens such as *Staphylococcus*, *Enterococcus*, *Salmonella*, and *Bacillus* vegetative cells and endospores, as well as non-pathogenic bacteria [12–18]. Some strains of *E. coli* have been widely tested for their susceptibility to CAPPs [10,19,20]. The susceptibility of *E. coli* strains is of particular relevance to our group because of our interest in urinary tract infections (UTIs) [21]. These strains are important because they are predominant causes of catheter-associated UTIs (CAUTIs) and because of their increasing multidrug resistance. Individuals who receive inpatient urinary catheterization or utilize outpatient intermittent urinary catheterization are at risk for CAUTIs. Intermittent catheters for outpatient use are sterilized and sealed during manufacturing and are intended as single-use devices (SUDs). Despite this intended use, SUD catheters are often reused, especially in low-income populations in developed countries and the developing world. Some clinicians have also advised the reuse of catheters [22,23]. Therefore, cleaning and disinfecting or sterilizing SUD catheters is important to reduce the introduction of pathogenic microbes into the bladder. Accepted methods for sterilization and high-level disinfection of devices (autoclave, ethylene oxide, glutaraldehyde, etc.) used by manufacturers and in hospitals are neither practical nor safe for home use. SUD catheters that individuals reuse are generally cleansed by rinsing with saline solution, washing with mild antibacterial soap and hot water, or treatment with harsh chemicals such as bleach or alcohol [22,24,25]. These methods may not be effective against microbes or biofilms within the lumens of the catheters. In fact, biofilms, especially drug-resistant biofilms, are a challenge in healthcare settings because they are notoriously difficult to eliminate [26–28]. Many antimicrobial chemicals used to inhibit bacteria in their free-floating planktonic state are rendered ineffective against biofilm due to the impenetrable protection barrier provided by the biofilm extracellular polymeric substance (EPS) matrix [26–30].

Also, any hydrophilic or antimicrobial coatings placed on SUD catheters during manufacturing [31] can be eroded by the use of harsh chemicals. The deleterious effects of these chemicals on the environment should also be considered as a rationale for why these reuse methods are unsustainable [32].

In this feasibility study, we aimed to establish whether UPEC and drug-resistant strains of *E. coli* are susceptible to the Ar-CAPP bactericidal action and to determine whether CAPP can be propagated through narrow flexible tubing mimicking urinary catheters while also retaining bactericidal activity.

## Materials and methods

### Bacterial strains and growth

*E. coli* strains were grown as previously described [33]. Briefly, overnight cultures of non-pathogenic strain MG1655, model UPEC strains DS17, CFT073, and UTI89, and multidrug resistant extended-spectrum ß-lactamase producing (ESBL) *E. coli* isolates ESBL41, ESBL146, ESBL168, and ESBL193 (from the Sysoeva lab culture collection) were grown in lysogeny broth (LB). Cells were collected by centrifugation and suspended in fresh LB. Cell suspensions were diluted so that 100 µL contained cell numbers sufficient for spread on a 10 cm diameter (78 cm^2^ area) Petri dish at initial surface densities of 10^2^, 10^4^, or 10^6^ cells per cm^2^ (S1 Fig in S1 File). After exposure to CAPP, plates were incubated overnight at 37°C.

### Production of cold atmospheric pressure plasma (CAPP)

**Fig 1:**
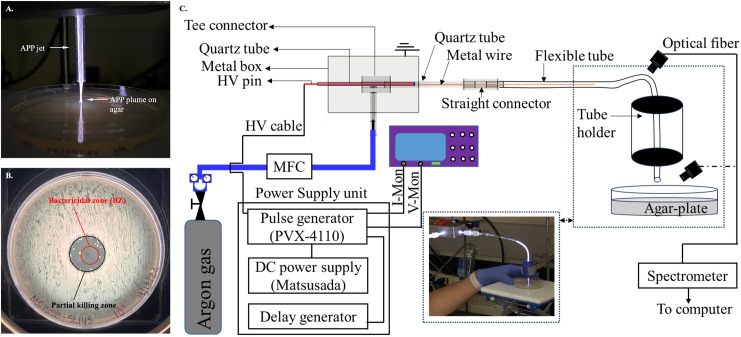
Experimental design for plasma treatment of *E. coli* and cleaning urinary catheters. **(A)** Sterilization by Ar-CAPP jet-treatment of bacterial cells on agar plate. **(B)** Bactericidal zone post-treatment by Ar-CAPP. **(C)** Schematic of Ar-CAPP propagating inside the tube and contacting surface inoculated media agar.

Generation of Ar-CAPP was done using the setup as previously described [33] with some modifications. Briefly, an unshielded configuration of the plasma jet consists of a ¼” Teflon Tee fitting as the central connector between the gas inlet, plasma outlet, and powered electrode. The jet outlet is a 2.90 mm ID and 6 mm OD quartz tube. Argon was the working gas and the flow rate was varied from 0.5 to 3 SLPM using an electronic mass flow controller. Setup 1 was mounted vertically so that the plasma plume emerged from a quartz tube with its impact perpendicularly directed onto the agar surface (Fig 1A–1B).

Setup 2 (Fig 1C) was intended to model urinary catheters with flexible Tygon tubing of internal diameters (IDs) 0.8, 1.5, 2.5, or 3.5 mm to cover the most widely used pediatric, adult female, and adult male catheters’ range (S1 Table in S1 File) [34].The top ends of all flexible tubes were connected to the outlet of the plasma jet quartz tube (inner diameter, ID = 2.90 mm, outer diameter, OD = 6 mm) using a straight Swagelok compression union. The total length of all flexible tubes was 24 cm. The tubing was flexed 90 degrees to direct the plasma jet output vertically to impact the agar surface. A metal wire was inserted on the inside of the quartz tube and the beginning of the flexible tube to extend the length of the plasma. This stainless-steel wire covered a length of ~5 cm in all flexible tubes and acted as a secondary powered electrode. At a unipolar pulsed DC voltage of 8 kV, frequency 6 kHz and pulse width 1 μs, the plasma extended to a length of ~16–18 cm, consistent with the length of commonly used adult female catheters [35–37].

### Measuring bactericidal action of CAPP

All plates contained 25 mL LB agar, and the outflow point of the plasma jet was 12 mm above the agar surface to eliminate any variability there. Bacterial culture with known cell density was loaded onto agar plates and then exposed to Ar-CAPP. After incubation to grow out the surviving bacteria, the plate was imaged. The image was then uploaded to the GNU Image Manipulation Program (GIMP) to measure the diameter of the bactericidal zone (Fig 1B) [21]. Experiments were done in technical triplicates that were repeated on three different days providing biological replicates. Means and standard deviations were calculated and shown for all plotted data.

**Fig 2:**
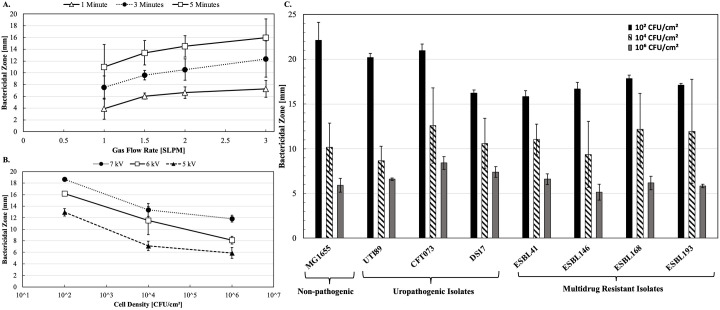
Quantifying bactericidal effects of cold Ar-CAPP against diverse *E. coli* strains. **(A)** Increasing the gas flow rate and time durations of exposure of cells yields more killing by cold Ar-CAPP when applying 6 kV. **(B)** Ar-CAPP bactericidal effects decreases at higher cell densities. **(C)** Triplicates of 10^2^, 10^4^, and 10^6^ CFU/cm^2^ MDR and UPEC isolates were exposed to cold Ar-CAPP with an applied 6 kV and 1.5 SLPM. A one-way ANOVA with post-hoc Dunnett’s test showed statistical significance *(p-value<0.05)* only for the ESBL41:MG1655 at 10^2^ CFU/cm^2^. All measurements were repeated at least 3 times with technical triplicates in each biological repeat. Error bars represent the standard deviation amongst the biological repeats.

**Fig 3:**
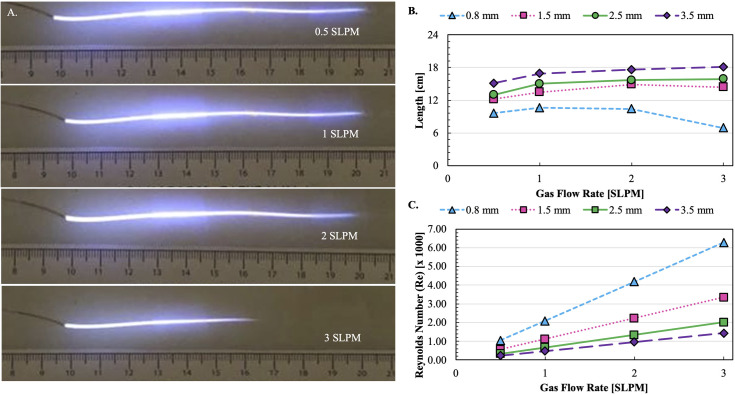
Ar-CAPP propagates through catheter-like tubing. **(A)** Propagation of plasma inside 0.8 mm flexible tube. **(B)** Lengths of the plasma plume inside flexible tubes of different IDs. **(C)** Reynold’s number (RN) variation inside tubes of different IDs.

**Fig 4:**
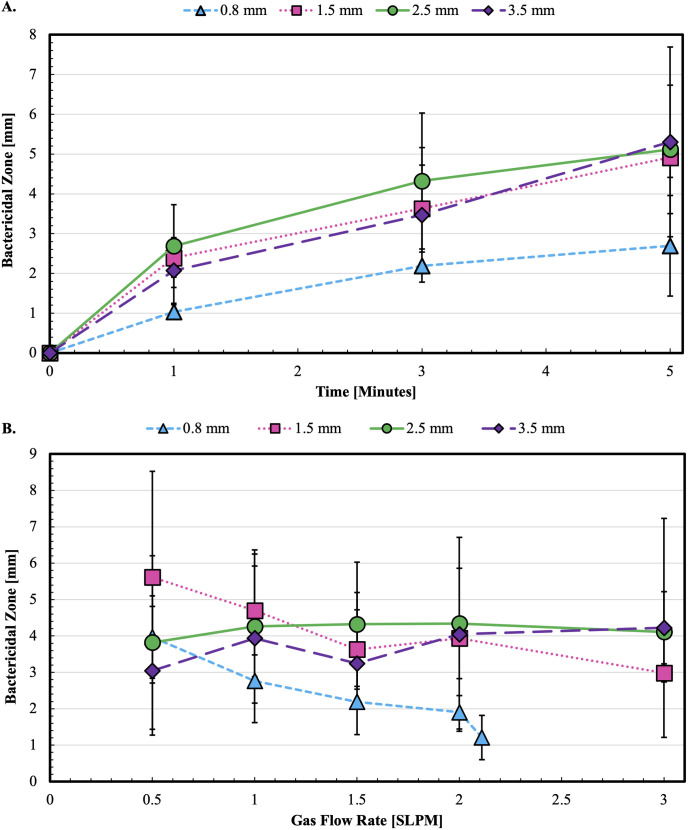
Ar-CAPP retains a bactericidal effect at the efflux point. **(A)** Evaluating bactericidal effect of cold Ar-CAPP flowing at 1.5 SLPM with an applied 8 kV over time. *E. coli* MG1655 at 10^4^ CFU/cm^2^ was the test organism. The first point represents no plasma treatment or control. **(B)** Effect of cold Ar-CAPP upon increasing gas flow rate. Bactericidal effect of tubing propagated Ar-CAPP is not increasing with increased gas flow, that is in contrast to unobstructed Ar-CAPP jet effect (Fig 2A).

Further experiments examined effects on bactericidal zones (BZ) of variables including time of exposure to plasma, gas flow rate, voltage used to produce plasma, *E. coli* surface cell density, and killing of various *E. coli* strains at fixed cell density, voltage, and gas flow rate (Figs 2A–2B and S3 Fig in S1 File). One-way ANOVA with Dunnett’s test for multiple comparisons was used to compare BZ size of each *E. coli* strain to BZ of the MG1655 control at different cell densities. In Setup 2, BZ were measured as in Setup 1 and analyzed as functions of tubing internal diameter, time of exposure to plasma, and gas flow rate (Figs 3 and 4).

### Measuring plasma characteristics

Electrical characteristics of the plasma were analyzed by measuring the voltage and current signals (Supporting Information file Section 1, S5 Fig in S1 File) while optical characteristics were analyzed using optical emission spectroscopy. The electrical signals were acquired at the output of PVX-4110 pulse generator using built-in current (I-mon) and voltage (V-mon) monitoring probes. To detect the presence of excited species, optical emission spectra inside and outside of the flexible tubing were acquired using a spectrometer (Model no. ULS4096CL-EVO, Avantes, Supporting Information file Section 2, S6–S8 Figs in S1 File). Reynolds numbers (RN) were calculated for different gas flow rates based on the ID of the flexible tubing, gas flow rate, and argon properties at atmospheric pressure and room temperature.

## Results

### CAPP killing multidrug-resistant uropathogens

A freshly spread confluent plate of *E. coli* cells was exposed to the output from an Ar-CAPP (Ar-CAPP) generator (Setup 1, Fig 1A). After overnight growth, a lawn of bacterial cells was seen with a central area devoid of growth surrounded by an area of sparse growth (Figs 1B and S1 Fig in S1 File). The central area was designated as the BZ. No viable cells were recovered when sampling the bactericidal zone. Absence of a strong effect of the underlying Petri dish geometry was shown by testing 4 different types of dishes (S2 Fig in S1 File).

BZ diameter increases with time of exposure at all gas flow rates tested, ranging from 1 to 3 standard liters per minute (SLPM) (Fig 2A). BZ diameter increases with increasing voltage over the range of 5–7 kilovolts and is greater with lower cell densities (Figs 2B and S3 Fig in S1 File). Fig 2B shows the results of plates seeded with 10^2^, 10^4^, or 10^6^ cells/cm^2^ and then treated with the same dose of Ar-CAPP. Ar-CAPP killing depended on bacterial surface density as the BZ was smaller with 10^6^ cells/cm^2^, compared to lesser densities. There was an approximately three-fold decrease in BZ as density increased from 10^2^ to 10^6^ CFU/cm^2^ (Fig 2C). Ar-CAPP treatment reduced this bacterial population by a factor of at least 10^6^.

UPEC models (UTI89, CFT073, DS17), ESBL *E. coli* clinical isolates (ESBL41, ESBL146, ESBL168, ESBL193), and control (MG1655) strain were all susceptible to Ar-CAPP killing regardless of surface cell density (Fig 2C). Susceptibility was greatest at 10^2^ cells/cm^2^ and least at 10^6^ cells/cm^2^. There were no statistically significant differences identified in pair-wise comparisons with a single exception of pair MG1655:ESBL41 at 10^2^ CFU/cm^2^.

### CAPP propagates through catheter-like tubing

The visible plasma plume within the flexible tubing propagated in the range of 60–180 mm (Figs 3A–3B and S4 Fig in S1 File). While the plume length typically increased with the increase of the Ar flow, the narrowest tubing with the ID of 0.8 mm showed different behavior (Fig 3A–3B). Measured plume length for 1.5-, 2.5-, and 3.5-mm ID tubing peaks in the 2–4 SLPM range with maximum lengths from 140 to 180 mm. Visible plasma plume length for 0.8 mm ID tubing is relatively constant over flow rates 0.5–2 SLPM and markedly shorter at 3 SLPM (Fig 3A and 3C). Failure of bacterial killing in tubing 0.8 mm ID beyond gas flow 2 SLPM was noted. This is attributed to the relatively higher gas turbulence inside the tube. Reynolds number (RN), a measure of laminar vs. turbulent flow [38] was calculated (Fig 3C). For the 0.8 mm ID tubing the RNs are greater than 2,000 for gas flow between 1 and 3 SLPM indicative of turbulent flow [39–41].

The effects of Ar-CAPP bactericidal action against *E. coli* MG1655 through catheter-like tubing are shown in Fig 4. BZ diameter increases with time, reaching approximately 5 mm at 5 minutes for tubing with IDs 1.5, 2.5, and 3.5 mm. BZ diameter for 0.8 mm ID tubing was less than 3 mm. BZs obtained with Ar-CAPP directly from the plasma jet (i.e., without flexible tubing) ranged from 10 to 16 mm after 5 minutes of exposure (Figs 2A–2B and S3A Fig in S1 File).

For large tubing IDs, the BZ diameter, at 2.5–3.5 mm, was relatively constant over gas flows ranging from 0.5 to 3 SLPM (Fig 4B). For the smallest tubing (0.8 mm ID), BZ diameters decreased as the flow rate increased and ceased altogether. In contrast, with Ar-CAPP directly from the plasma jet (Fig 2A) BZ increased with flow rates ranging from 1 to 3 SLPM; maximum BZ diameter was approximately 12 mm at 3 SLPM.

As we obtained bactericidal action at the outlet of the tubing, we also measured the formation of the reactive hydroxyl radical species using optical spectrophotometry to confirm that this species is formed within the tubing as well. Spectra obtained from inside the tubing space showed presence of the OH radicals at all tested gas flow rates and IDs (Supporting Information file, Section 2, S6–S8 Figs in S1 File).

## Discussion

The primary finding of this study is that Ar-CAPP retains bactericidal activity even after traversing a 24 cm length of flexible Tygon tubing which was used as a model for urinary catheters (Figs 3 and 4). Since the plasma plume emerging from the tubing was bactericidal, it is speculated that bactericidal plasma persisted through the entire tubing length. This speculation seems reasonable since the efflux plume was bactericidal and since OH radical was detected within the tubing. However, this speculation remains to be investigated.

Assay of plasma bactericidal action against agar-supported bacteria at fixed surface densities has been improved and semi-quantified by adaptation of the clearance zone method (Figs 1 and 2) [42,43]. Bactericidal power of Ar-CAPP was demonstrated by production of BZs on plates with initial cell densities as high as 10^6^ cells/cm^2^ (Fig 1). This should be regarded as a lower limit for bactericidal activity as it was the highest surface concentration tested. Decreasing BZ diameter with increasing cell surface densities suggests layered structures that shadow neighboring cells, thus increasing population resistance.

In the annulus immediately outside the BZ, isolated surviving colonies are seen (Fig 1B). This may be due to dissipation of bactericidal effects as the plasma plume spreads. Alternatively, but less likely, these survivors may be mutants with increased plasma resistance. Such survivors require further investigation.

The extent of bacterial killing as measured by BZ diameter depends on cell density (CFU/cm^2^) and plasma generator operating conditions (Fig 2). In general, BZ diameter increases with time of exposure, gas flow rate, and voltage. It decreases with increasing cell density (as previously discussed). These suggest parameters that could be optimized for real-world urinary catheter studies.

Clinical isolates of *E. coli* urinary pathogens, such as previously mentioned UPEC and ESBL MDR strains, possess virulence factors not found in non-pathogenic and susceptible *E. coli* strains [21,44]. Accordingly, these strains were tested for susceptibility to the bactericidal effects of Ar-CAPP (Fig 2C). All strains were susceptible to Ar-CAPP killing at all surface cell densities tested. With the exception of one ESBL strain at lowest density, no significant differences in susceptibility were observed comparing non-pathogenic MG1655 with UPEC and MDR strains. Thus, the presence of virulence factors and multiple drug resistance plasmids in clinical isolates neither protects nor sensitizes to Ar-CAPP killing, and such plasmas can be regarded as a general agent for killing *E. coli* strains.

Previously described experiments were done with direct exposure of seeded agar surfaces to the immediate output of an Ar-CAPP source. Whether plasma could propagate and remain bactericidal upon efflux from catheter-like tubing was investigated with a modified plasma-generating apparatus (Fig 4). This was possible (S4 Fig in S1 File) using catheter models of realistic length (24 cm) and internal diameters (0.8, 1.5, 2.5 and 3.5 mm). BZ diameters for all tubing increased with time of exposure (Fig 4A) but were relatively constant with increasing gas flow rate for all sizes except 0.8 mm, for which BZ diameter decreased with increasing flow rate and ceased above 2 SLPM. This latter result is concerning because it covers the size range for pediatric catheters [45]. Results in Fig 3 indicate that turbulent flow quenches the plasma plume at higher gas flow rates. Propagation of plasma through the entire length of 0.8 mm ID tubing might be approached through lower gas flow rates coupled with higher activation voltages.

This feasibility study is a preliminary laboratory study that has limitations. Tygon tubing was used as a model system, and other materials, including authentic urinary catheters, should be evaluated. Only a single bacterial species was tested, whereas lumens of real-world catheters may be contaminated with multi-species biofilms. Additionally, we did not assess CAPP bactericidal action on internal surfaces of bacteria-inoculated tubing and relied only on the plasma efflux bactericidal efficiency as an estimate. A biofilm model is needed. Future work will develop bacterial biofilms on the internal surfaces of tubing to determine whether Ar-CAPP will ablate biofilms inside narrow tubing. Novel plasma-generating systems have become available, including wide-area sheet plasma generators (K.G. Xu, personal communication) and multiple-ring plasma devices [46]. This latter device may make possible the simultaneous decontamination of both inner and outer surfaces of flexible tubing. Nevertheless, CAPP is a dry technology that overcomes issues associated with currently used wet chemical methods, which increases their usability and decreases environmental impact.

## Conclusions

This pilot study has shown that clinically relevant *E. coli* strains are susceptible to Ar-CAPP bactericidal action, and that Ar-CAPP propagated through catheter-dimensioned tubing retains bactericidal activity after emerging from the distal end of the tubing. This study can serve as a basis for further development of this technology for disinfection and sterilization of medical insertion devices where re-use was impossible, ineffective, or unsustainable.

## Supporting information

S1 File This PDF file includes supporting figures S1-S8, S1 Table, and two sections describing experimental measurements of Ar-CAPP source and plasma parameters. (PDF)

S1 Data Excel tables with the experimental data and results of statistical tests. (XLSX)
