# Supplementary material for: Propagation of antibacterial cold atmospheric pressure plasma through small-bore tubing
Source: PLoS One. 2025 Aug 21;20(8):e0328375. doi: 10.1371/journal.pone.0328375 (PMC12370019; doi:10.1371/journal.pone.0328375)
Supplement: S1 File — (PDF) [file pone.0328375.s001.pdf]

# Supporting Information

## Propagation of antibacterial cold atmospheric pressure plasma through small-bore tubing

Elanie F. Briggs[1], Bhagirath Ghimire[2], John A. Mayo[1], Maryellen S. Kelly[3,4], Kunning G. Xu[2], Tatyana A. Sysoeva[1]
[1]Department of Biological Sciences, The University of Alabama in Huntsville, Huntsville, AL
[2]Department of Mechanical and Aerospace Engineering, The University of Alabama in Huntsville, Huntsville, AL
[3]Healthcare of Women and Children Division, School of Nursing, Duke University, Durham, NC
[4]Department of Urology, Duke University, Durham, NC

**Table of Contents:**

**S1 Figure. Method to test different bacterial cell densities (CFU/cm$^2$).**
**S1 Table. Internal diameters of frequently used straight catheters.**
**S2 Figure. Analysis of Ar-CAPP bactericidal effect on bacterial cells loaded on Petri dishes of various geometry.**
**S3 Figure. Quantitative analysis of Ar-CAPP bactericidal effect on bacterial cells loaded on agar surface.**
**S4 Figure. Plasma plume propagation in Tygon tubing past transporting wire.**
**Section 1. CAPP source characterization.**
**S5 Figure. Current and voltage waveforms of plasma source.**
**Section 2. Spectroscopic measurements.**
**S6 Figure. Optical emission spectra were recorded from the plasma propagating inside flexible tube.**
**S7 Figure. Variation within the hydroxyl radical-specific peak intensity when measured inside the tubing.**
**S8 Figure. Variation within the hydroxyl radical-specific peak intensity when measured outside of the tubing.**

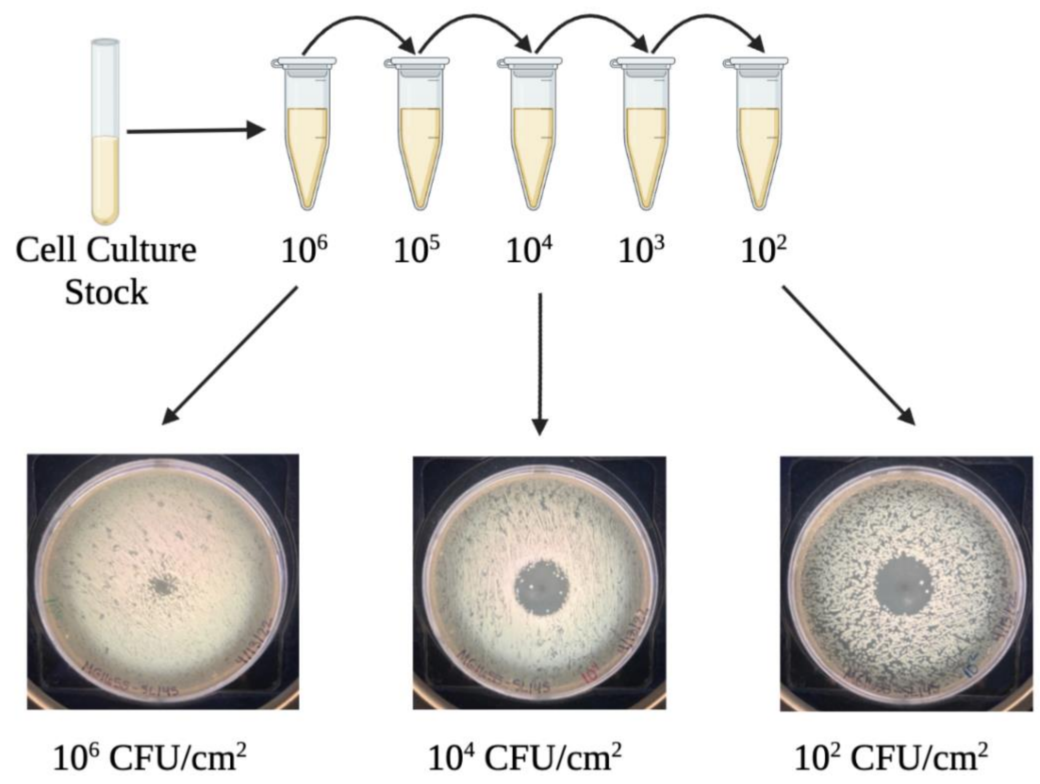


**S1 Figure. Method to test different bacterial cell densities (CFU/cm$^2$).**

**S1 Table. Internal diameters of frequently used straight catheters.**

| Catheter Gauge | OD calculated, mm | ID measured, mm | Catheter type |
|---|---|---|---|
| 6Fr | 2.00 | 1.2 | Coloplast Speedi |
| 8Fr | 2.67 | 1.6 | Coloplast Speedi |
| 10Fr | 3.33 | 1.9 | BD hydro |
| 10Fr | 3.33 | 2 | Coloplast Speedi |
| 12Fr | 4.00 | 2.8 | Coloplast speedi |
| 14Fr | 4.67 | 3.1 | Coloplast Speedi |
| 14Fr | 4.67 | 2.64 | Coloplast |
| 14Fr | 4.67 | 2 | unknown |
| 16Fr | 5.33 | 3.5 | Glide |
| 16Fr | 5.33 | 3.16 | GentleCath |
| 16Fr | 5.33 | 3.2 | unknown |
| 18Fr | 5.67 | 3.71 | BARO All Purpose |
| 20Fr | 6.94 | 2.93 | Bardex I.C. |
| 22Fr | 8.62 | 3.58 | Bardex I.C. |
| 26Fr | 8.08 | 4.47 | Bardex I.C. |

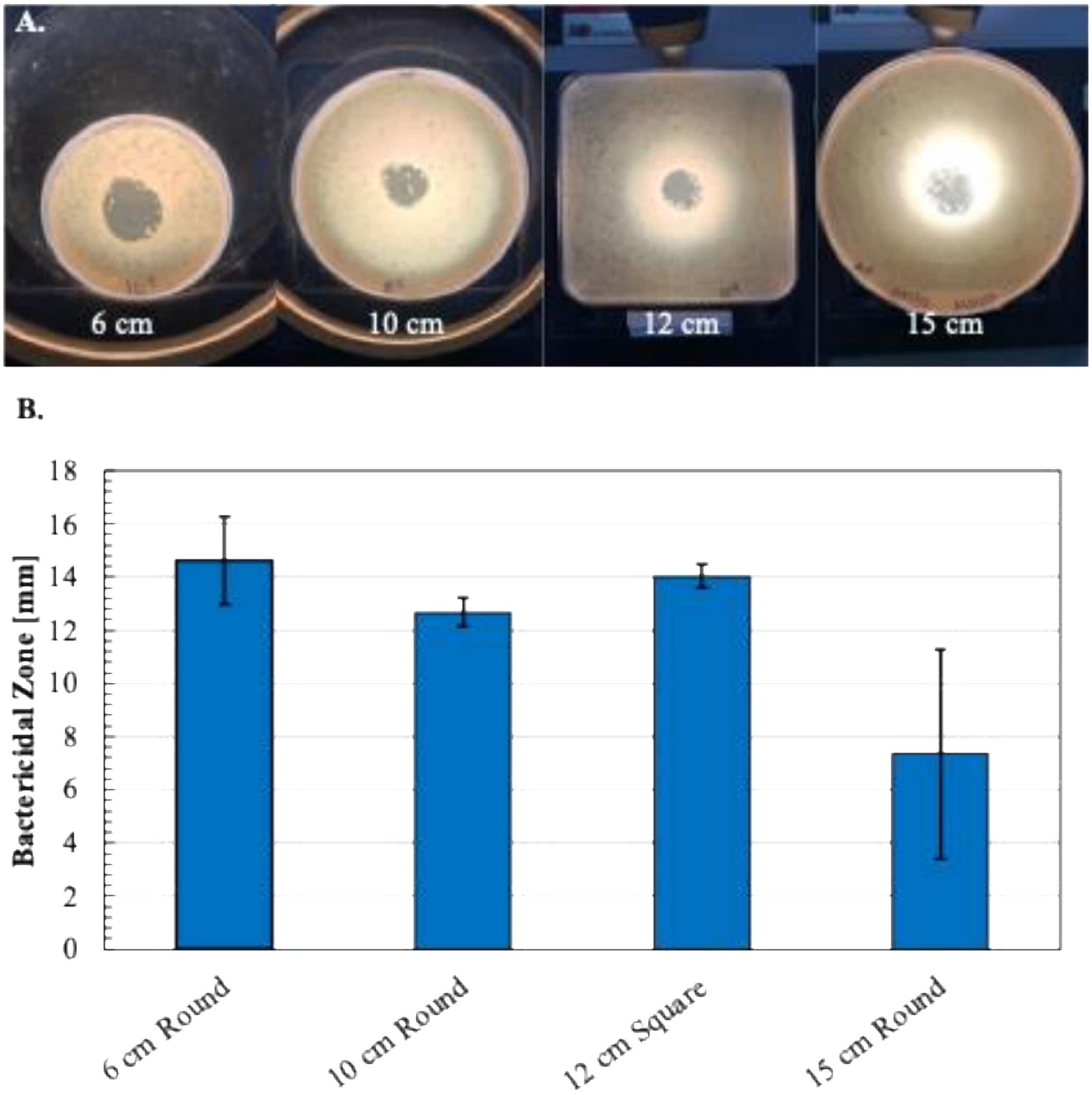


**S2 Figure. Analysis of Ar-CAPP bactericidal effect on bacterial cells loaded on Petri dishes of various geometry.** (A) Inoculated *E. coli* MG1655 at $10^4$ CFU/$cm^2$ on media agar was exposed to Ar-CAPP at 6 kV and 1.5 SLPM for 3 minutes. (B) Bactericidal zone diameter measurements post-treatment with CAPP.

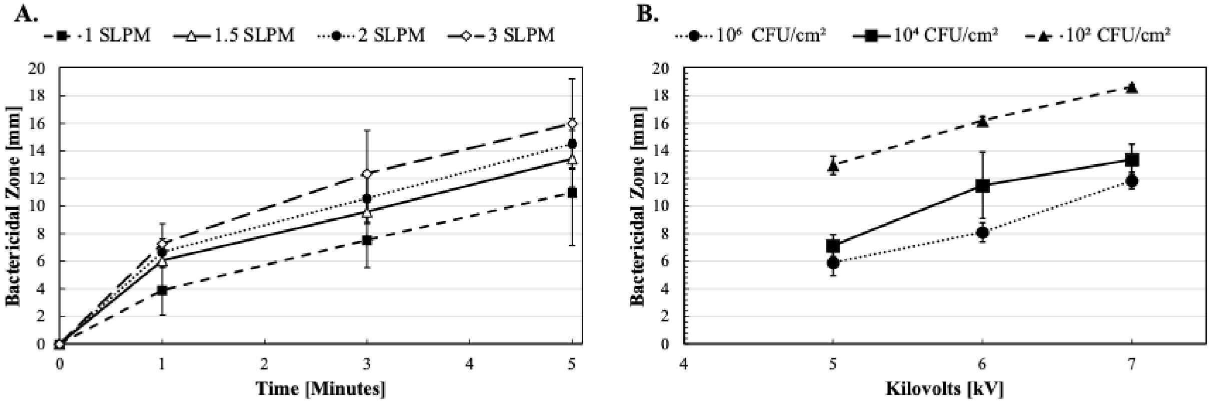


**S3 Figure. Quantitative analysis of Ar-CAPP bactericidal effect on bacterial cells loaded on agar surface.** (A) Increased time durations for exposing bacterial isolates to cold Ar-CAPP generates more killing. The first point represents no plasma treatment. (B) Increasing the voltage yields more bactericidal action by the Ar-CAPP for each cell density.

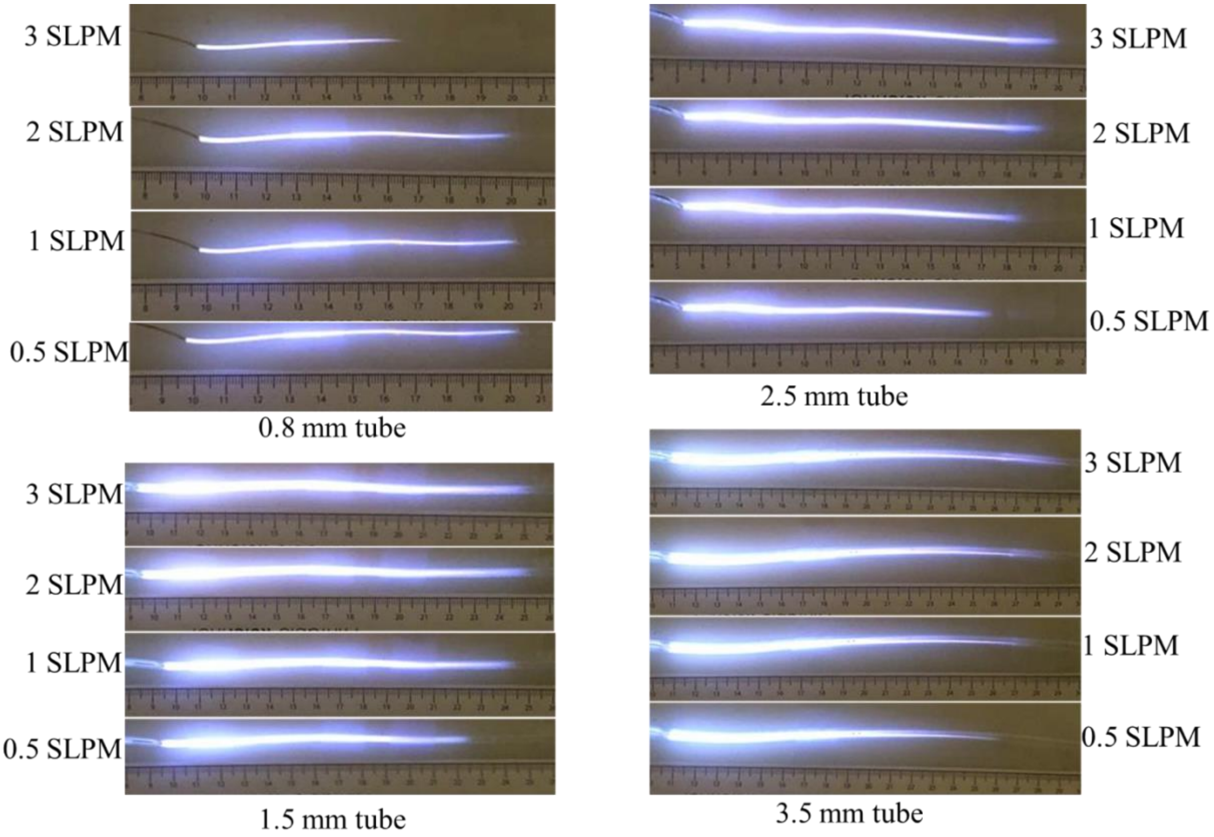


**S4 Figure. Plasma plume propagation in Tygon tubing past transporting wire.** Plume length in 0.8-3.5 mm ID tubing was measured and plotted in Fig 3B.

**Section 1. CAPP source characterization.**

S5 Figure shows the electrical waveforms of the plasma jet measured at the high voltage electrode. Plasma was generated at an applied voltage: 8 kV, frequency: 6 kHz and pulse width: 1 μs. The positive discharge current peak in S5 Figure appears due to the accumulation of wall charges on the inner walls of the quartz tube during the rising part of the applied voltage. These charges are reversed in polarity during the falling edge of the applied voltage giving rise to negative discharge current peak. The average energy per pulse ($E_{avg}$) was computed as:

$$E_{avg} = V\int Idt,$$

where V is the DC applied voltage and $\int Idt$ represents the magnitude of accumulated charges during the rising (or falling) edge. The average energy per pulse was measured to be 3.56 mJ and it resulted in the power deposition of ca. 21.41 W for Ar flow rate of 1.5 SLPM. These values were almost constant for other gas flow rates used in the study. This is attributed to the minimal change in neutral density as also observed in our previous study [1].

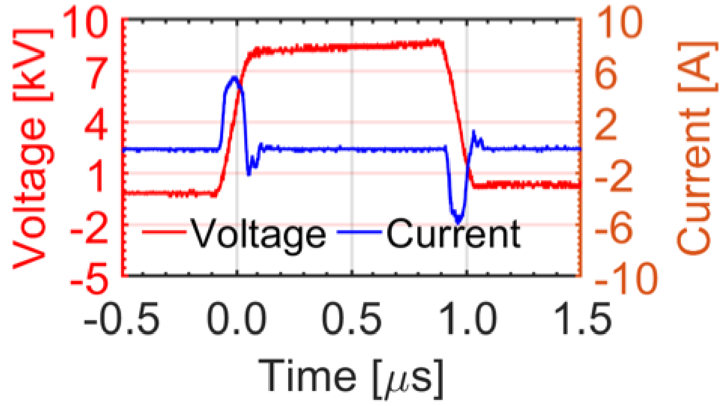


**S5 Figure. Current and voltage waveforms of plasma source.** Current and voltage waveforms were measured from the output of PVX-4110 pulse generator at Ar flow rate of 1.5 SLPM.

**Section 2. Spectroscopic measurements.**

We conducted the optical emission spectroscopy inside of the flexible tube and the results are presented in S6-S8 Figures. The emissions below 700 nm (S6 Fig) is mainly composed of hydroxyl radicals (OH) at 309 nm. The intensity from OH was very low as compared to major emissions in the visible region of the spectrum. This is because, the Tygon tubes used in this study have low transmission in the UV range and a relatively high integration (100 ms) had to be used to record the spectra. Emissions from OH takes place from the dissociation of water molecules which could be present in the feeding gas as an impurity. We speculate that this could be an important species to play a role in the decontamination of bacteria inside the flexible tubes. The emission intensity from OH was dependent on the ID of the flexible tube and Ar flow rate. These results are summarized in S7 Figure. For 0.8 mm tube, the intensity from OH continuously decreased with increasing gas flow rate. For 1.5 mm and 2.5 mm tubes, the intensity reached maximum at Ar flow rates of 1 and 2 SLPM, respectively, and started to decline afterwards. Similarly, for the 3.5 mm tube, the intensity reached a maximum at 1.5 SLPM after which it attained constant values. These trends in the change in OH intensities are ascribed to the effects caused by differences in gas flow velocities and turbulence at different positions of the tube (Fig 3C). The relative change in intensities for different IDs of the flexible tubes and gas flow rates are summarized in S8 Figure.

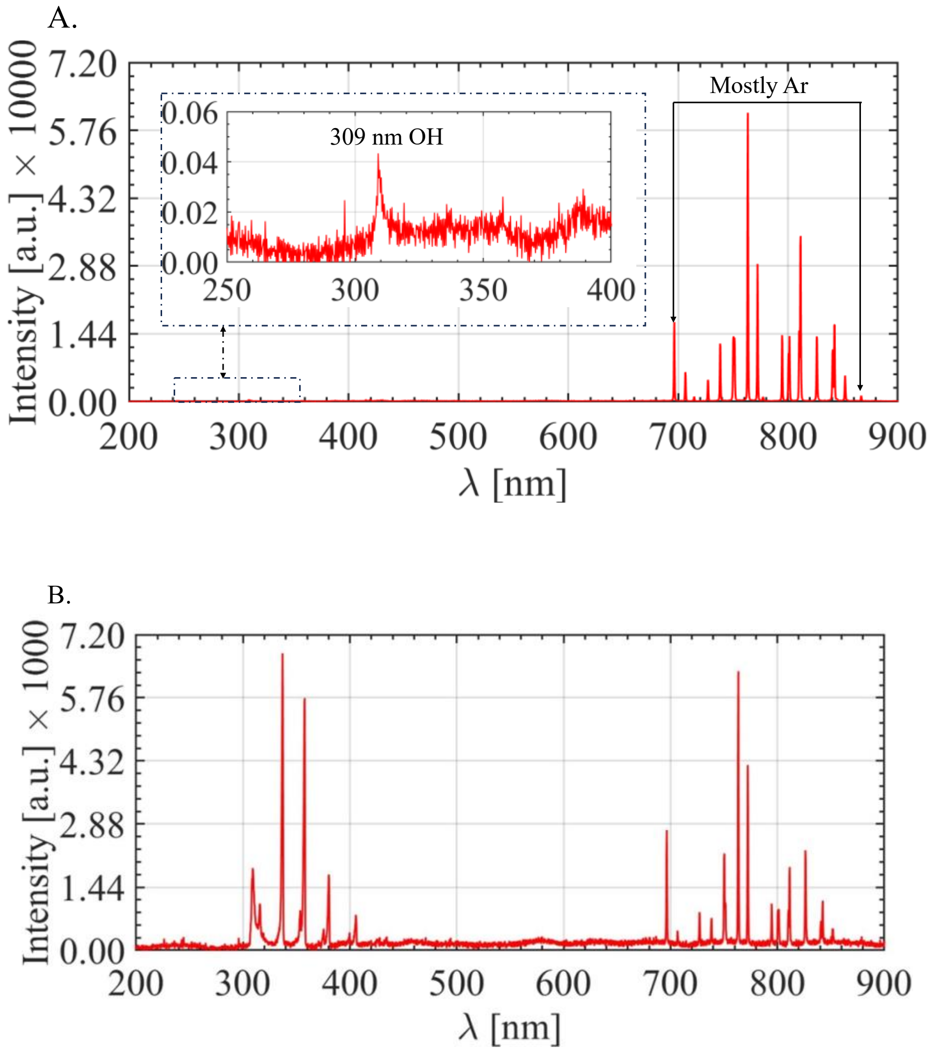


**S6 Figure. Optical emission spectra were recorded from the plasma propagating inside flexible tube**. (A) Spectrum inside the 1.5 mm ID tubing at 5 cm below the transporting electrode. (B) Spectrum outside of the 1.5 mm tubing.

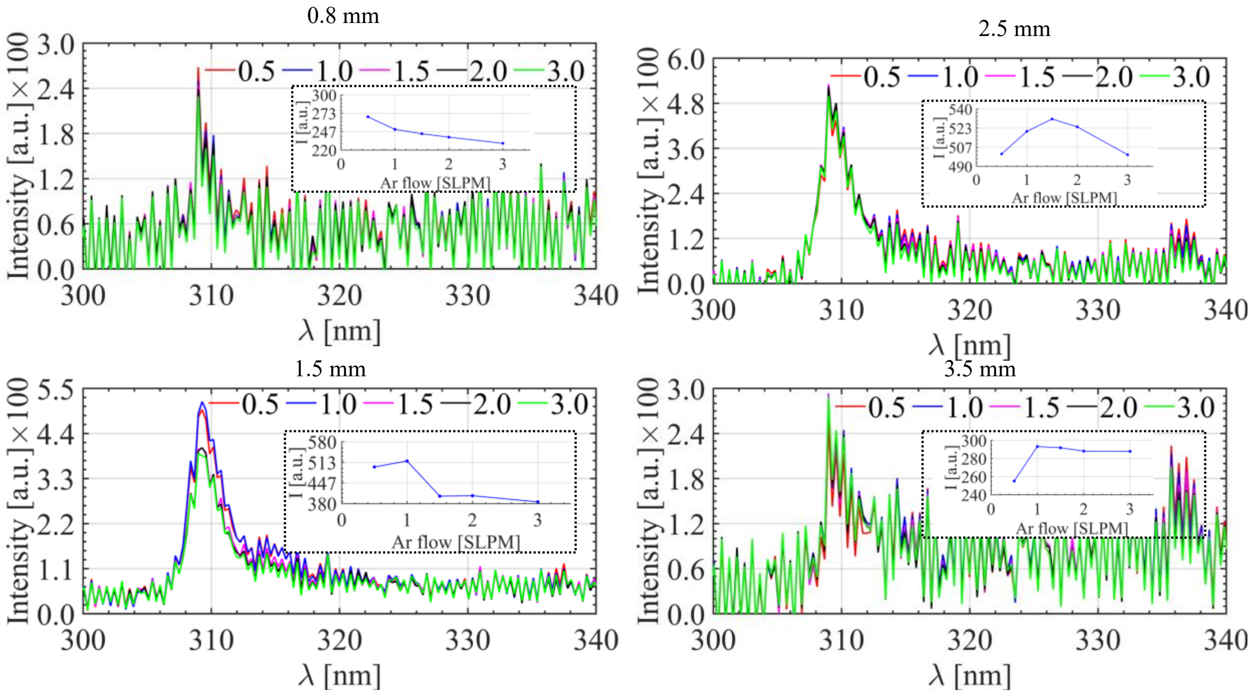


**S7 Figure. Variation within the hydroxyl radical-specific peak intensity when measured inside the tubing.** Absorption spectra around the characteristic 309 nm wavelength are shown for four different ID tubings (0.8-3.5 mm) with overlays from five different Ar flow rates (0.5-3 SLPM). Insets plot intensity at 309 nm at different gas flow rates.

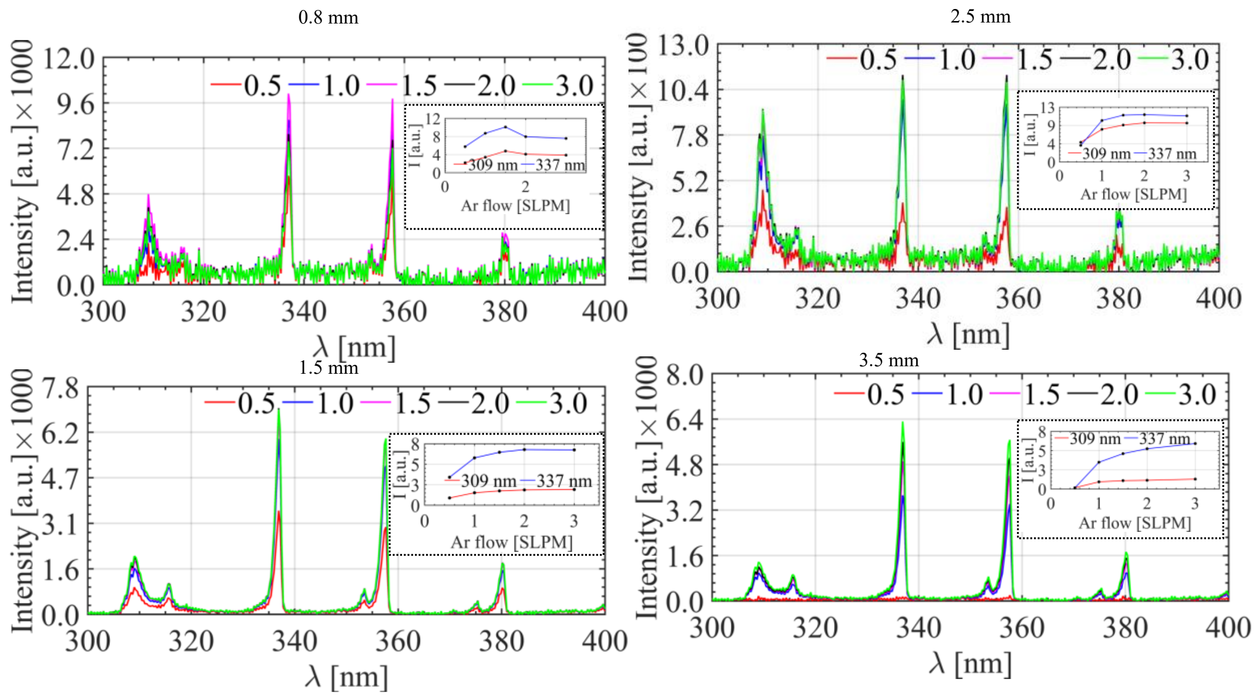


**S8 Figure. Variation within the hydroxyl radical-specific peak intensity when measured outside of the tubing.** Absorption spectra between 300 and 400 nm wavelength are shown for four different ID tubings (0.8-3.5 mm) with overlays from five different Ar flow rates (0.5-3 SLPM). Insets plot intensity at 309 and 337 nm at different gas flow rates.

**References:**

1. Gott RP, Xu KG (2019) OH production and jet length of an atmospheric-pressure plasma jet for soft and biomaterial treatment. IEEE Trans Plasma Sci 47(11):4988–4999.
